# FGF23, alpha-Klotho and vitamin D mediated calcium-phosphate metabolism in haemodialysis patients

**DOI:** 10.5937/jomb0-27408

**Published:** 2021-03-12

**Authors:** Ozge Tugce Pasaoglu, Ayse Senelmis, Ozant Helvaci, Ulver Derici, Hatice Pasaoglu

**Affiliations:** 1 Gazi University, Faculty of Health Sciences, Department of Nutrition and Dietetics, Ankara, Turkey; 2 Gazi University, Faculty of Medicine, Department of Medical Biochemistry, Ankara, Turkey; 3 Gazi University, Faculty of Medicine, Department of Internal Medicine, Section of Nephrology, Ankara, Turkey

**Keywords:** calcium-phosphate metabolism, FGF23, haemodialysis, vitamin D, Klotho, metabolizam kalcijum-fosfata, FGF23, hemodijaliza, vitamin D, Klotho

## Abstract

**Background:**

Klotho is a prote˝in that acts as a co-receptor for FGF23. FGF23-Klotho axis has great importance regarding the regulation of mineral metabolism by kidneys. In this study, we analysed FGF23, Klotho, 1,25-dihydroxyvitamin D3, 25-hydroxyvitamin D, parathormone, Calcium and Phosphate levels of haemodialysis patients in order to investigate the nature of the mineral metabolism disruption in chronic kidney diseases.

**Methods:**

Sixty haemodialysis patients and 34 healthy controls were included in the study. Serum iFGF, cFGF, and soluble Klotho were analysed using ELISA kits. Moreover, 1,25-dihydroxyvitamin D3 was determined using LCMS/MS. Calcium, phosphate, iPTH and 25-hydroxyvitamin D were measured using autoanalyzers.

**Results:**

In haemodialysis patients, iFGF23, cFGF23, iPTH and P levels were significantly higher, and 1,25-dihydroxyvitamin D3, Klotho and Ca levels were significantly lower compared with the control group. There was no significant difference in the 25-hydroxyvitamin D levels.

**Conclusions:**

Our study showed that lack of sufficient amounts of Klotho is crucial for mineral metabolism disruptions seen as a complication of chronic kidney diseases. Despite the high levels of the hormone, FGF23 is unable to accomplish its function properly, likely due to deteriorated kidney function in haemodialysis patients.

## Abstract (sr)

**Uvod:**

Klotho je protein koji deluje kao koreceptor za FGF23. Osa FGF23-Klotho ima veliku važnost u regulaciji metabolizma minerala u bubrezima. U ovom istraživanju analizirali smo nivoe FGF23, Klotho, 1,25-dihidroksivitamina D3, 25-hidroksivitamina D, parathormona, kalcijuma i fosfata kod pacijenata na hemodijalizi kako bismo istražili prirodu poremećaja metabolizma minerala u hroničnim bubrežnim bolestima.

**Metode:**

U istraživanje je uključeno šezdeset pacijenata na hemodijalizi i 34 zdrave osobe u kontrolnoj grupi. Serumi iFGF, cFGF, i rastvorljivi Klotho su analizirani korišćenjem ELISA kompleta. Korišćenjem LC-MS/MS određen je 1,25-dihidroksivitamin D3. Kalcijum, fosfat, iPTH i 25-hidroksivitamin D mereni su korišćenjem autoanalizatora.

**Rezultati:**

Kod bolesnika na hemodijalizi nivoi iFGF23, cFGF23, iPTH i P su bili značajno viši, a nivoi 1,25-dihidroksivitamina D3, Klotho i Ca bili su značajno niži u poređenju sa kontrolnom grupom. Nije bilo značajne razlike u nivoima 25-hidroksivitamina D.

**Zaključak:**

Istraživanje je pokazalo da je nedostatak dovoljnih količina Klotho presudan za poremećaje metabolizma minerala koji se smatraju komplikacijom hroničnih bolesti bubrega. Uprkos visokom nivou, hormon FGF23 nije u mogućnosti da pravilno obavlja svoju funkciju, verovatno zbog pogoršane funkcije bubrega kod pacijenata na hemodijalizi.

## Introduction

The kidney is one of the most important regulatory organs of mineral metabolism, including calcium (Ca) and Phosphate (P). Disorders of mineral metabolism can be seen in chronic kidney diseases (CKD). CKD-mineral and bone disorder (CKD-MBD) has been described as a systemic disease with ectopic calcifications and abnormalities in mineral and bone metabolism as a complication of CKD [1]. CKD-MBD is one of the complications affecting mortality and morbidity in CKD patients [2]. The serum Ca levels are regulated by two hormones which are parathormone (PTH) and 1,25-dihydroxyvitamin D3 (1,25(OH)_2_D3, Calcitriol). These hormones regulate Ca levels while affecting serum Phosphate levels [3]. Hyperphosphatemia is also an important complication that increases cardiovascular disease in CKD [4]. When investigating hyperphosphatemic conditions, it was found that some factors other than these calcitrophic hormones were also involved in the regulation of serum phosphate [3]. FGF23 was first described to have phosphaturic effects in hypophosphatemic rickets and tumour-induced osteomalacia. Currently, FGF23 is known to be the regulating hormone in phosphate metabolism. FGF23 is produced primarily by osteocytes and osteoblasts in bone tissue and binds to the FGF23-αKlotho receptor complex [5]. FGF23 is available in 3 major forms in blood: intact FGF (iFGF), which is the mature and active fulllength form, and inactive forms resulting from the cleavage of iFGF, containing amino-terminal peptide segment (nFGF) and carboxyl-terminal peptide segment (cFGF) [6].

Regardless of the causes of CKD, the main pathophysiological event is a gradual decrease in the number of functional nephrons [7]. This reduction in nephron number activates the FGF23-αKlotho endocrine axis [8]. Phosphate excretion per nephron should be increased to maintain phosphate balance unless dietary phosphate intake is reduced. This demand is also met by an increase of iFGF23. Calcitriol decreases as a result of increased FGF23, and the decrease in calcitriol is followed by an increase in PTH. Clinical studies have revealed that an increase in iFGF23, a decrease in calcitriol, and an increase in PTH are seen in the same order during the development of CKD. The serum Phosphate levels increase last [9] [10]. When the residual nephron count decreases to a level that cannot meet the dietary phosphate level and urine excretion, cardiovascular complications, ectopic calcifications and CKD-MBD associated with hyperphosphatemia occur in end-stage renal disease [10]. iFGF23 also acts by suppressing synthesis and accelerating degradation of 1,25(OH)_2_D3. This is achieved by reducing the mRNA expression of 1α-hydroxylase, which converts 25-hydroxyvitamin D (25(OH)D) to 1,25(OH)_2_D3, and increases 25-hydroxyvitamin D 24-hydroxylase levels involved in the degradation of vitamin D [11].

Meanwhile, cFGF also acts by inhibiting iFGF23-αKlotho signal axis and urinary phosphate excretion as a result of its binding to FGFR-αKlotho complex [6].

The members of the FGF family are bound to the FGFR receptors. While the affinity of iFGF23 for FGFRs is normally low, the αKlotho protein acting as a co-receptor increase the affinity of iFGF23 to the αKlotho-FGFR complex [12] [13]. Klotho was first described as the gene of genetically mutated mice with the same name showing signs of premature ageing [14]. After the discovery of other paralogs such as Klotho and Klotho, this Klotho protein was named αKlotho to differentiate from these paralogs. αKlotho protein has two forms: membrane-bound αKlotho and soluble αKlotho (sKl). sKl is formed by the cleavage of full fragment αKlotho protein, and it functions in circulation, cerebrospinal fluid, urine and distant organs. The FGF23-αKlotho pathway plays a major role in regulating phosphate and vitamin D metabolism [15]. However, there are still unresolved questions in the FGF23 mechanism. For example, Klotho is primarily expressed in renal distal tubules, parathyroid gland, and choroid plexus, and, as well as the FGF23, the αKlotho signalling pathway was first identified in the distal tubules, but the exact function of this pathway in the proximal tubules was not fully elucidated [5]. In addition, iFGF and cFGF increase together when CKD progresses, but there are not enough studies to reveal the role of cFGF in phosphate mechanism [6].

The aim of this study was to compare the serum levels of iFGF23, cFGF23, intact PTH (iPTH), 1,25(OH)_2_D3, 25(OH)D, Ca, P and sKl in patients with CKD undergoing haemodialysis with the control group and to reveal the pathophysiological role of these molecules in CKD.

## Materials and Methods

This study was approved by Zekai Tahir Burak Women's Health Training and Research Hospital Ethics Committee (approval number: 12/2017). Informed consent was obtained from all individual participants included in the study.

Sixty haemodialysis patients treated and followed up at our Department of Nephrology were included in the study as a haemodialysis (HD) group. The patients included in the HD group were 36 males, age 48.22±10.71 years, and 24 females, age 42.00±11.02 years. Thirty-four age-matching healthy individuals, who have no existing or no history of chronic health conditions, were selected as the control group. The mean age of 18 males in the control group was 41.22±6.34, and the mean age of 16 females was 41.88±8.24.

From all individuals, blood samples taken after 8-10 hours of fasting were centrifuged, and serums were separated. While the Ca, P, iPTH, 25(OH)D parameters were analysed on the same day, in order to measure the 1,25(OH)_2_D3, iFGF23, cFGF23 and sKl levels, the serums were aliquoted and stored at -80°C until analysis. The serum iFGF23, cFGF23 and sKl levels were analysed using the ELISA kits (SunRed Biological Technology, Shanghai, China). The serum levels of 1,25 (OH)_2_D3 were determined by LC-MS/MS using the ImmuChrom (Heppenheim, Germany) brand kit. The serum Ca, and P levels were measured using the Beckman Coulter AU5800 Clinical Chemistry System autoanalyser. The serum levels of iPTH and 25(OH)D were measured by the Beckman Coulter DxI 800 Access Immunoassay System autoanalyser.

Statistical analysis was performed using the SPSS Statistics 20 software. The data were reported as the mean ± SD and median (minimum-maximum). Student's t-test was used for comparisons between groups that showed normal distribution, and the Mann Whitney U test was used for parameters that were not normally distributed. According to parameter suitability, Pearson's or Spearman's correlation tests were used for correlation analyses. The value P<0.05 was considered statistically significant.

## Results

The mean levels (with standard deviations) and median levels (with minimum and maximum) of iFGF23, cFGF23, 1,25(OH)_2_D3, sKl, 25(OH)D, iPTH, Ca, P of 60 haemodialysis patients and 34 subjects in the control group were depicted in Table 1. Compared with the control group, the serum iFGF23, cFGF23, iPTH and P levels were significantly higher (*P*<0.01 for all) in haemodialysis patients and the serum 1,25(OH)_2_D3, sKl and Ca levels were significantly lower (*P*<0.01 for all) in haemodialysis patients. There was no significant difference in serum 25(OH)D levels between haemodialysis and control groups (*P*=0.151).

**Table 1:** Comparison of serum iFGF23, cFGF23, 1,25(OH)_2_D3, sKl, 25(OH)D, iPTH, Ca and P levels between haemodialysis and control groups ^1^Mann-Whitney U Test; ^2^Student’s t-test

	Hemodialysis Group (n=60)		Control Group (n=34)		
	Mean ± SD	Median (min - max)	Mean ± SD	Median (min - max)	P
iFGF23 (ng/L)	352.16 ± 155.62	342.83 (86.52 - 736.52)	155.06 ± 73.41	119.68 (52.39 - 361.74)	<0.011
cFGF23 (ng/L)	246.33 ± 108.51	248.98 (55.41 - 515.1)	122.61 ± 71.13	98.98 (42.4 - 321.92)	<0.011
1,25(OH)_2_D3 (ng/L)	27.02 ± 8.74	26.45 (3.7 - 49.4)	48.42 ± 12.13	46.4 (26.8 - 71.5)	<0.012
sKl (µg/L)	1.94 ±1.11	1.86 (0.14 - 6.31)	4.32 ± 3.18	3.53 (0.26 - 12.18)	<0.011
25(OH)D (µg/L)	13.86 ± 7.40	12 (5.78 - 40)	16.40 ± 10.00	13.54 (7 - 59.6)	0.1511
iPTH (ng/L)	417.77 ± 347.33	311.85 (31.7 - 1595.2)	45.76 ± 25.61	38.05 (3.41 - 109.1)	<0.011
Ca (mg/L)	89.4 ± 6.7	89 (66 - 106)	94.3 ± 3.8	93.8 (85.4 – 102.2)	<0.011
P (mg/L)	43.8 ± 10.2	45 (21.7 - 64)	35.4 ± 5.0	35.2 (25.4 – 46.2)	<0.012

**Figure 1:**
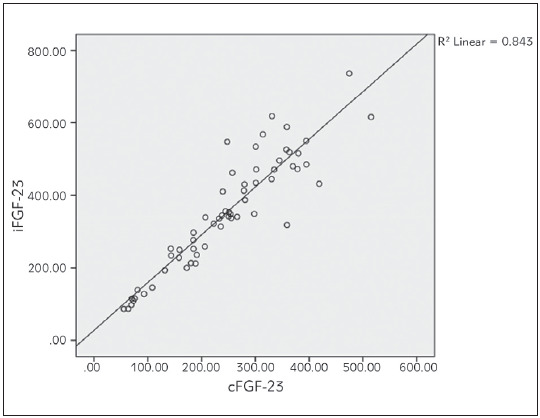
Correlation between iFGF-23 and cFGF-23 in haemodialysis patients.

**Figure 2:**
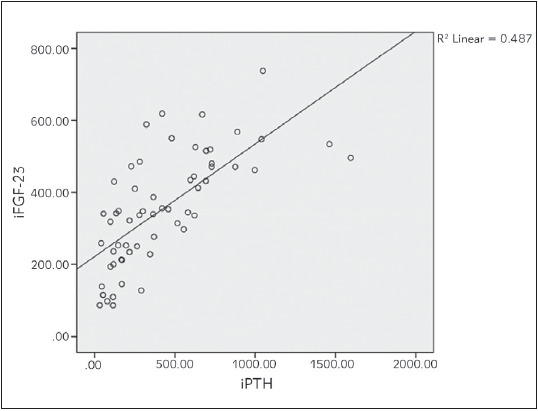
Correlation between iFGF-23 and iPTH in haemodialysis patients.

**Figure 3:**
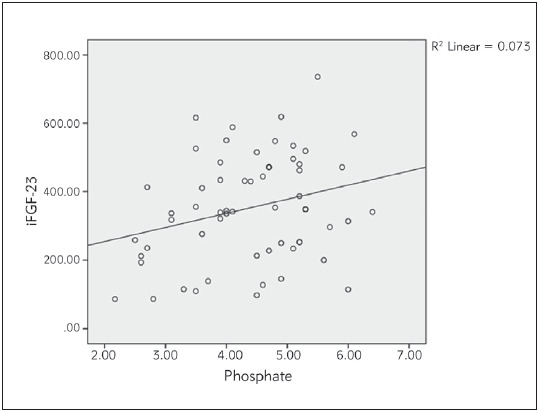
Correlation between iFGF-23 and Phosphate in haemodialysis patients.

**Figure 4:**
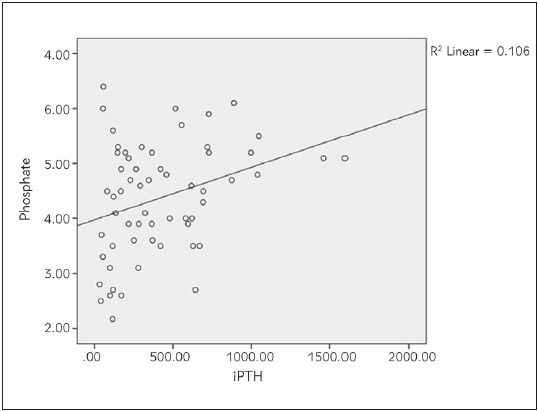
Correlation between Phosphate and iPTH in haemodialysis patients.

Bivariate correlation analyses were conducted to determine relationships between the iFGF23, cFGF23, 1,25(OH)_2_D3, sKl, 25(OH)D, iPTH, Ca and P levels in haemodialysis patients Table 2. Strong positive correlation was found between iFGF23 and cFGF23 (P<0.01, p=0.000, r=0.918). iFGF23 was also positively correlated strongly with iPTH (P<0.01, p=0.000, r=0.772). There was a weak positive correlation between iFGF23 and P levels of haemodialysis patients (P<0.05, p=0.036, r=0.271). There was also weak positive correlation between iPTH and P levels (P<0.05, p=0.010, r=0.328) (see Figure 1, Figure 2, Figure 3, Figure 4). Between other parameters of haemodialysis patients, no significant relationship was found in bivariate correlation analyses.

**Table 2:** Correlations between parameters in haemodialysis patients. ^1^Pearson’s correlation test ^2^Spearman’s correlation test

		1,25(OH)2D3	iFGF23	cFGF23	sKl	25(OH)D	iPTH	Ca	P
1,25(OH)2D3	*p*	-	0.917^1^	0.932^1^	0.230^1^	0.915^2^	0.786^2^	0.066^2^	0.489^1^
	r	-	0.014	0.011	0.157	-0.014	-0.036	0.239	0.091
iFGF23	*p*		-	0.000^1^	0.593^1^	0.146^2^	0.000^2^	0.401^2^	0.036^1^
	r		-	0.918	-0.070	-0.190	0.772	-0.111	0.271
cFGF23	*p*			-					
	r			-					
sKl	*p*				-	0.375^2^	0.523^2^	0.502^2^	0.345^1^
	r				-	0.117	-0.084	0.088	0.127
25(OH)D	*p*					-	0.174^2^	0.715^2^	0.868^2^
	r					-	-0.178	0.048	0.022
iPTH	*p*						-	0.822^2^	0.010^2^
	r						-	0.030	0.328
Ca	*p*							-	0.376^2^
	r							-	-0.116
P	*p*								-
	r								-

## Discussion

Causes of elevated FGF23 levels in CKD patients have been investigated for many years. In addition to the theory that renal clearance of FGF23 decreases as CKD progresses, end-organ resistance to phosphaturic action of FGF23 due to the deficiency of αKlotho protein, a cofactor protein of FGF23, has been emphasised in recent years [11]. Koh et al. [16] found a significant decrease in mRNA expression αKlotho in biopsies performed in patients with CKD. Higher FGF23 levels in CKD patients are one of the physiological compensatory mechanisms to stabilise the increased serum P levels as the number of intact nephrons decreases. FGF23 ultimately induces phosphaturia and lowers the serum P levels. Furthermore, FGF23 inhibits the expression of 1α-hydroxylase and inhibits the conversion of 25(OH)D to its active metabolite, 1,25(OH)_2_D3 [17]. Also, there are studies suggesting that FGF23 increases PTH secretion and gene expression in in vivo animal models, the PTH levels in CKD patients may be increased in response to hypocalcemia due to suppressed 1,25(OH)_2_D3 and may be explained by hyperplasia in parathyroid as a result of secondary hyperparathyroidism to maintain the Ca levels by Casensitive receptors [18] [19]. In our study, in parallel with these findings, the serum iFGF23, cFGF23, iPTH and P levels in haemodialysis patients were significantly increased and the 1,25(OH)_2_D3, sKl and Ca levels were significantly decreased when compared with the control group. Probably due to FGF23 inhibiting 1α-hydroxylase activity, the 1,25(OH)_2_D3 levels decreased, but the 25(OH)D levels did not change significantly.

The most important protein in this pathophysiological model is αKlotho protein. It has been shown in studies on CKD as a result of glomerulonephritis, nephrotoxicity, diabetic and hypertensive nephropathy characterised by renal cell loss that αKlotho mRNA is downregulated and the sKl levels decrease in serum and urine [11]. In a study conducted by Pavik et al. [20] on eighty-seven CKD patients in different stages, it was reported that while the PTH and P levels increased in the later stages of CKD, the plasma sKl levels firstly decreased then FGF23 increased and 1,25(OH)_2_D3 decreased. In a study by Sakan et al. [21], it was also shown that the sKl levels were significantly reduced in early stages of CKD, but the serum P levels remained within the normal range. In a study by Isakowa et al. [9] on 3879 patients in the Chronic Renal Insufficiency Cohort Study, in early stages of renal disease, while the serum phosphate and parathormone levels were within the normal range, the FGF23 levels were significantly increased compared to a healthy population, and as the disease progressed, the increase in FGF23 became more significant, and hyperphosphatemia and secondary hyperparathyroidism developed. These results indicate that FGF23 is increased to compensate for P retention caused by renal insufficiency in the early stage of CKD patients, and managed to maintain the P levels within the normal levels since the sKl levels were not yet sufficiently reduced. However, as renal damage progresses, because of the resistance to FGF23 and decreasing αKlotho expression, the P and FGF23 levels increase, and the sKl levels decrease, and as a response to these changes, the PTH levels increase and Ca levels decrease [11]. In our study investigating the mineral metabolism in end-stage renal disease, it was revealed that the sKl, 1,25(OH)_2_D and Ca levels decreased and FGF23, PTH and P levels increased in haemodialysis patients.

Although as GFR progressed, the increased FGF23 levels and decreased sKl levels were demonstrated in many studies and compared with healthy controls, different significant positive or negative correlations between FGF23, sKl and other hormones and minerals were found in different studies. In a cohort study conducted by Kim et al. [22] between 2006 and 2011, on 243 CKD patients, the sKl levels were significantly decreased while the FGF23 levels were increased in advanced CKD stages. They also found that the sKl levels correlated positively with GFR and negatively with the logFGF23, logPTH, P and Ca levels. Liu et al. [6] also divided 152 CKD patients according to their stages and revealed that iFGF23, cFGF23/sKl ratio and the serum P levels were significantly increased in stage 3-5 and the serum Klotho and Ca levels were decreased. They also found that P was positively correlated with iFGF23, cFGF23, and the cFGF23/sKl ratio and cFGF23 was positively correlated with iFGF23 and Ca. In another study on 100 haemodialysis patients with cardiological diseases, log(FGF23) was correlated negatively with log(sKl) and positively with log(P), and log(sKl) was positively correlated with log(25(OH)D) and log(1,25(OH)_2_D) [23]. In a study conducted by Rotondi et al. [24] on 68 CKD patients, sKl negatively correlated with PTH and P and positively correlated with Ca, and FGF23 positively correlated with PTH and P. In contrast to all these studies, Seiler et al. [25] followed 312 CKD patients with stage 2-4 for an average of 2.2 years and claimed that the sKl levels were not significantly correlated with GFR and other hormones or minerals in calcium-phosphate metabolism, and high FGF23 levels had no prognostic effect on sKl. In our study, we also found that iFGF23 was positively correlated with cFGF23, iPTH, and P and iPTH was also positively correlated with the P levels.

Many researchers view CKD as a state of FGF23 resistance caused by the lack of αKlotho. We also support this hypothesis as our results showed high levels of iFGF23, cFGF23, iPTH and Phosphate, and low levels of sKl, 1,25(OH)_2_D3 and Ca in the study group. Despite the high levels of the hormone, FGF23 is unable to accomplish its phosphaturic function properly to lower plasma phosphate levels, quite likely due to the lack of proper kidney function in HD patients. However, further studies are recommended to confirm the correlations between hormone and minerals in mineral metabolism of CKD patients with the findings of our and previous studies.

## List of abbreviations

1,25(OH)_2_D3, 1,25-dihydroxyvitamin D3, Calcitriol; 25(OH)D, 25-hydroxyvitamin D; cFGF23, Carboxyterminal fibroblast growth factor-23; CKD, Chronic kidney disease; CKD-MBD, Chronic kidney disease-mineral and bone disorder; FGF23, Fibroblast growth factor-23; HD, Haemo-dialysis; iFGF23, Intact fibroblast growth factor-23; iPTH, Intact parathormone; sKl, soluble Klotho.

## Acknowledgements

This study was supported by Gazi University Scientific Research Projects Unit (Project code: 01/2017-17).

## Conflict of interest statement

The authors stated that they have no conflicts of interest regarding the publication of this article.
